# Anapole-assisted giant electric field enhancement for surface-enhanced coherent anti-Stokes Raman spectroscopy

**DOI:** 10.1038/s41598-021-90061-5

**Published:** 2021-05-20

**Authors:** Maryam Ghahremani, Mojtaba Karimi Habil, Carlos J. Zapata-Rodriguez

**Affiliations:** 1 Photonics Research Laboratory, Center of Excellence for Applied Electromagnetic Systems, University of Tehran, North Kargar Ave., Tehran, Iran; 2 Faculty of Physics, University of Tabriz, 51664 Tabriz, Iran; 3 Department of Optics and Optometry and Vision Science, University of Valencia, 46100 Burjassot, Spain

**Keywords:** Optics and photonics, Applied optics, Integrated optics

## Abstract

The coherent anti-Stokes Raman spectroscopy (CARS) techniques are recognized for their ability to detect and identify vibrational coherent processes down to the single-molecular levels. Plasmonic oligomers supporting full-range Fano-like line profiles in their scattering spectrum are one of the most promising class of substrates in the context of surface-enhanced (SE) CARS application. In this work, an engineered assembly of metallic disk-shaped nanoparticles providing two Fano-like resonance modes is presented as a highly-efficient design of SECARS substrate. We show that the scattering dips corresponding to the double-Fano spectral line shapes are originated from the mutual interaction of electric and toroidal dipole moments, leading to the so-called non-trivial first- and second-order anapole states. The anapole modes, especially the higher-order ones, can result in huge near-field enhancement due to their light-trapping capability into the so-called “hot spots”. In addition, independent spectral tunability of the second Fano line shape is exhibited by modulating the gap distance of the corner particles. This feature is closely related to the electric current loop associated with the corner particles in the second-order anapole state and provides a simple design procedure of an optimum SECARS substrate, where the electric field hot spots corresponding to three involved wavelengths, i.e., anti-Stokes, pump, and Stokes, are localized at the same spatial position. These findings yield valuable insight into the plasmonic substrate design for SECARS applications as well as for other nonlinear optical processes, such as four-wave mixing and multi-photon surface spectroscopy.

## Introduction

Interaction between the surface charge-density oscillations of noble-metal nanoparticles and the incident photons at the resonant frequency leads to the coherent and collective oscillation of free electrons confined at the metal-dielectric interfaces, which refers to localized surface plasmon resonances (LSPRs)^1–3^. The plasmon-enhanced light-matter interaction can confine light beyond the diffraction limit^1,4–6^. This remarkable feature makes the structure well-suited for a plethora of optical elements and applications^7–9^.

Under some particular configurations of metallic nanostructures, the coupling of elementary plasmon modes may lead to the generation of so-called Fano-like resonance^10–12^, which can be identified by its asymmetric line shape and steep profile. The phenomenon was originally observed in atomic systems where a continuum experiences quantum-mechanical interference with a discrete localized state^13^. In plasmonic structures, the Fano resonance is generally attributed to the spectral overlap of a broad symmetric super-radiant (bright) mode and a narrow asymmetric sub-radiant (dark) mode through the near-field hybridization^14^, where constructive and destructive interferences occur in a very narrow spectral window. The bright mode is typically referred to any highly-radiative resonant mode that can couple directly to an incident plane-wave. On the contrary, dark plasmon modes cannot be directly excited by the incident light due to their vanishing radiative mode^15–17^. This results in strongly suppressed far-field scattering and purely trapped modes. It is noted that a dark mode does not exist in the single noble-metal nanoparticles with regular shapes such as spheres^18^. Breaking the geometrical symmetry^18–21^, heterogeneous configuration^22–24^, structural nano-assembly^25^, phase retardation^26^, and the polarization direction of the incident light^27^ are the most important parameters which can enable efficient excitation of the dark modes and hybridization of plasmons of different multipolar symmetry. The narrower spectral linewidth compared to the standard plasmon resonances as well as the large local field enhancement provided by the dark modes, make the plasmonic Fano-based structures an ideal platform for diverse applications such as refractive index chemical and biological sensing^24,28–30^, surface-enhanced spectroscopy^31,32^, low-threshold nano-lasers^33^, and novel on-chip photonic device designs^34,35^.

Metallic nanoparticle clusters in the form of the trimer^36^, quadrumer^30^, pentamer^24^, heptamer^37^, and higher-order^25^ assemblies are one of the most promising ways to generate Fano-like resonance line shapes. The structural and chemical characteristics of the clusters such as size, shape, thickness, and material of the particles present in the cluster play an important role to obtain the desired Fano response. In addition, inter-particle gap distances between the adjacent particles define the coupling strength between LSPR modes of the elementary nano-scatterers.

In terms of the analysis of such atypical line profiles in oligomer plasmonic structures, several different techniques have been conducted over the recent years. Plasmon hybridization theory^38^ has been extensively exploited to intuitively describe the interactions in plasmonic systems, where the structure under consideration is subdivided into two or more subsystems with known properties. The modes of each subsystem are added constructively or destructively to form a bonding (symmetric) and an anti-bonding (anti-symmetric) mode^39^, leading to the formation of Fano-like resonance line shape. In addition, multiple studies have been devoted to theoretically investigate and interpret the origin of these anomalous resonances in metallic oligomers, such as the coupled mass oscillator model^40–42^,the coupled-mode formalism^43^, the circuit model of Fano resonance^44^, and the rigorous quantum electro-dynamic formulation^45,46^. These simplistic methods, however, are inadequate to provide a complete scenario of Fano-like spectral profiles in plasmonic oligomers, forcing the use of full-wave theories^45,46^.

Regarding the application, plasmonic Fano-based oligomer structures have been of particular interest to act as a substrate for single molecular detection as well as vibrational imaging. In this context, Halas et al.^47^ demonstrated that a plasmonic quadrumer substrate supporting a Fano-like resonance mode can be applied to acquire single-molecule detection sensitivity using the coherent anti-Stokes Raman spectroscopy (CARS)^48,49^. CARS is a specific type of four-wave mixing^50^ (FWM) non-linear optical process where two laser beams and a Stokes field coherently interact through the third-order susceptibility of the identified material to get access vibrational properties. To achieve an efficient Raman signal enhancement in a plasmonic SECARS substrate depends on simultaneous enhancement of electric field at the pump, Stokes, and anti-Stokes frequencies as well as having hot spots with the same spatial positions^51^. This indicates individual contribution of each of the three wavelengths in SECARS enhancement factor (EF). He et al.^51^ presented a plasmonic asymmetric trimer assembly supporting double-Fano line shapes which brings all of the three CARS frequencies hot spots to the same spatial location. The achieved EF for SECARS signal in this structure is around $2 \times {10^{11}}$ for the gap distance of $g = 12\,{\text{nm}}$. Recently, Zhang et al.^52^ theoretically investigated the plasmonic crisscross dimer exhibiting an electric dipole bright mode and magnetic dipole dark modes, which can be matched with the wavelength of pump, Stokes and anti-Stokes lights. Their proposed design achieves a significant amplification of the CARS signal reaching near a factor of ${10^{13}}$ for the 8-nm gap size.

Extending the aforementioned idea, we propose and theoretically analyze a metallic octamer structure representing double-Fano spectral line profile, which is constructed by an assembly of thin-film disk-shaped nanoparticles. Compared to the similar proposals based on multi-disks CARS substrates^53^, our design enables efficient generation of a single hot spot governing the CARS emission through a symmetrically centered nanogap. Our theoretical study provides an in-depth analysis of scattering dips in plasmonic oligomers, and their corresponding electric current distribution gives insight to design an optimum substrate for SECARS applications. To begin with, the Cartesian decomposition method in the finite element based commercial software (COMSOL Multiphysics) is exploited to calculate the contribution of the five leading multipoles in the total scattering cross-section (SCS). Decomposition results reveals that the two characteristic dips originate from a destructive interference between electric and toroidal^54–56^ dipole moments, indicating the first- and second-order anapole states^57^ excited in the system. The effects of the structure size as well as the illumination angle on the evolution of Fano line shapes is then explored. These analyses provide a simple design procedure for an optimum multi-resonance SECARS substrate. In this regard, independent spectral tunability of the first-order anapole state is exhibited by modulating the gap distance of the corner particles. Benefiting from such an independent tunability behavior, three excited resonance modes with large field enhancements can be achieved whose hot spots are spatially overlapping and their wavelengths spectrally match with the wavelengths of pump, Stokes, and anti-Stokes beams. When the inter-disk gap spacing is reduced to $g= 8\,{\text{nm}}$, the near-fields are further enhanced due to the improved energy confinement, and the maximum SECARS EF is approaching to ${10^{15}}$. Therefore, it is expected that the proposed oligomer design could be useful for the applications based on biosensing and nonlinear surface spectroscopy.

## Results and discussion

### Double-Fano resonant structure

**Figure 1:**
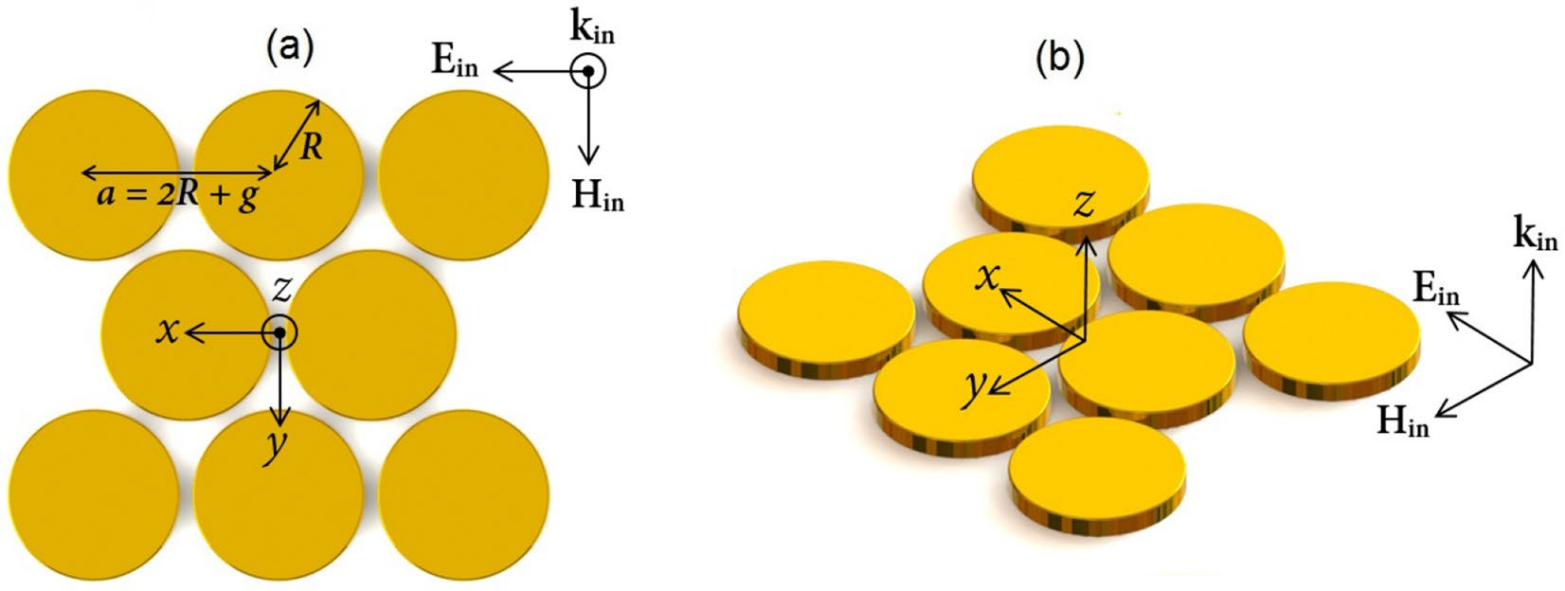
Schematic diagram of our proposed oligomer supporting the double-Fano spectral line profile (**a**) 2D and (**b**) 3D views. The structure is a finite triangular lattice of free-standing disk-shaped gold nanoparticles with lattice constant ${a} = 180\,{\text{nm}}$, where all the particles have the radius ${R} = 84\,{\text{nm}}$, height ${h} = 20\,{\text{nm}}$, and gap distance of ${g} = 12\,{\text{nm}}$. This oligomer is illuminated by an *x*-polarized plane wave propagating along the *z*-direction. The electric field is parallel to the axis of the central dimer, denoted as the parallel polarization.

Let us first propose a simple plasmonic oligomer which serves as a basis for our engineered SECARS substrate. The structure presented here is a finite triangular lattice of disk-shaped gold nanoparticles as shown in Fig. 1. The geometry has lattice constant $a = 180\,{\text{nm}}$ and disk radius $R = 84\,{\text{nm}}$ which is equivalent to the gap distance of $g = 12\,{\text{nm}}$. The height of the disk-based structure is always kept constant at $h = 20\,{\text{nm}}$, which has little influence on the scattering spectrum of the oligomer. Here, similar to Ref.^58^, we have focused on relatively thin gold films that can be realized benefiting from advances in lithography and self-assembly techniques^59^. In terms of the shape of the particles, nano-disks have several advantages such as easy fabrication, polarization-independent property, and relatively simple control of parameters. Note that although the near field enhancement considerably increases with the reduction of the gap size, the gap distance is not considered sub-10-nm to avoid the proximity effects that deteriorates the field enhancement due to nonlocal response^60^. This undesired effect is due to the significant localization of electromagnetic fields caused by the coupling between the adjacent nanoparticles^61^. The dispersive relative permittivity of gold is taken from the experimental data in Ref.^62^. For simplicity, the surrounding dielectric environment is assumed to be a vacuum $\left( {{\varepsilon _d} = 1} \right)$ in all simulations. According to the point made in Ref.^51^, introduction of the dielectric substrates or probe molecules in practice only shifts the scattering dips to longer wavelengths accompanied with a slight increase in linewidth because of dielectric screening. Apparently, deposing the particles on top of a metallic substrate results in the improvement of the performance due to the contribution of image modes^63^.

**Figure 2:**
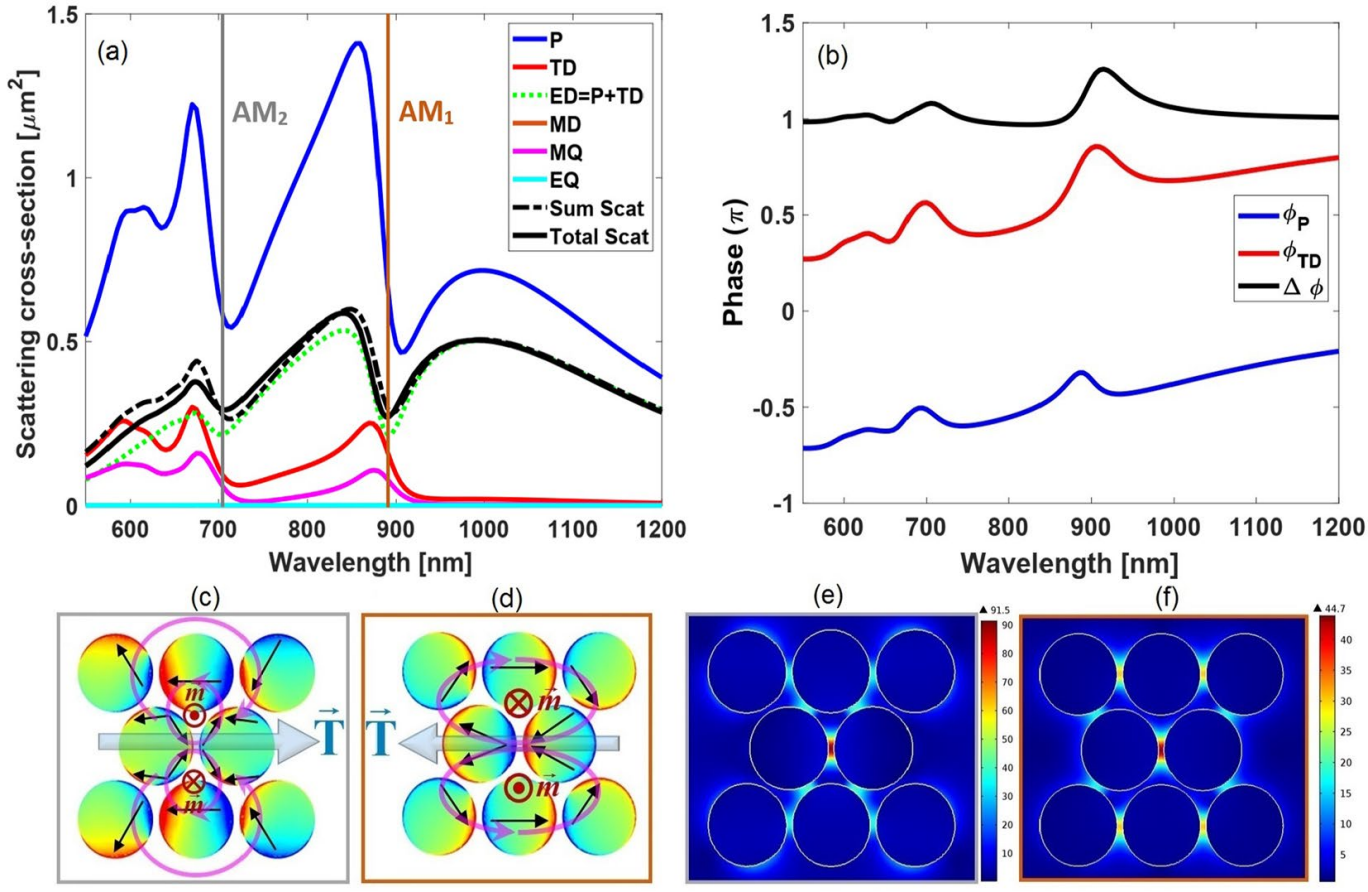
(**a**) The simulated SCS spectra for our proposed oligomer as well as contributions of the five dominant multipole moments to the total radiated field. “Sum Scat” states for the SCS as the sum of the multipole contributions;“Total Scat” states for the total SCS calculated directly in COMSOL (See “Computational methods”). The labels P, MD, EQ, MQ, and TD denote electric dipole, magnetic dipole, electric quadrupole, magnetic quadrupole, and toroidal dipole modes, respectively . Also, $AM_1$ and $AM_2$ represent the so-called first- and second-order anapole states with characteristic wavelength, respectively . (**b**) The phases of the electric dipole ${\phi _P}$ and toroidal dipole ${\phi _{TD}}$ moments as well as their difference $\Delta \phi$ versus wavelength. The surface charge density distribution is shown at (**c**) $AM_2$ wavelength and (**d**) $AM_1$ wavelength. Distribution of electric field amplitude $\left( {{{\left| \mathbf{E} \right| } \big / {\left| {\mathbf{E_{inc}}} \right| }}} \right)$ in the mid-height plane of the oligomer at (**e**) the second- and (**f**) first-order anapole modes. The SCS derived from ED results as the coherent superposition of P and TD contributions, symbolically represented as P+TD.

The total SCS spectrum of our proposed structure is shown in Fig. 2. This scattering spectrum exhibits two characteristic dips at the wavelengths of ${\lambda _1} = 705\,{\text{nm}}$ and ${\lambda _2} = 890\,{\text{nm}}$ representing excitation of the Fano-like resonance modes. To get some insight into the observed Fano line shapes, Cartesian multipolar analysis is implemented through the following paragraphs.

### Theoretical investigation of asymmetric double-Fano resonance mode

Generally, when an incident plane wave interacts with a nanoparticle, the re-emitted beam at an observation point located in the far-field zone can be described in terms of radiation from different multipoles. To identify the contribution of each multipole, we exploit decomposition of the total induced fields inside the meta-molecule under-study into approximate Cartesian multipole basis including contributions of the toroidal dipole moments. In the framework of approximate Cartesian decomposition, the contribution of each multipole moment in the total radiation of any particle with arbitrary shape can be calculated by starting from the induced electric currents and then simplifying the special functions with the small argument asymptotic terms in the long-wavelength regime^64^ (See Methods).

The multipolar SCS is computed to elucidate the nature of the different optical resonances sustained by the structure, particularly those regard the Fano-like resonance line shapes. Figure 2 includes contribution of the five leading order multipole moments in the total SCS spectrum and their summation obtained by Eq. (17) (See “Methods”). Here, contribution of the Cartesian electric and magnetic dipoles are represented by P and MD, electric and magnetic quadrupoles are denoted with EQ and MQ, respectively, and the toroidal dipoles are labeled by TD. As can be seen in Fig. 2a, the sum of Cartesian multipole contributions is in a good agreement with directly calculated total SCS (See “Computational methods”) throughout most parts, except for the region with shorter wavelength. The small discrepancy between these spectra can be explained by the long-wavelength approximations made in Cartesian multipole decomposition^64,65^. To further illustrate, we have employed the multipole expansion method in spherical coordinate, showing the results for the first four modes as well as their summation (See Fig. S1 in Supplementary Information). We see in Fig.2a that the most contributive multipoles to the meta-molecule response are the electric dipole as well as closely matched toroidal dipole and magnetic quadrupole responses. Furthermore, the total interference of the Cartesian electric dipole P and the toroidal dipole TD moments (represented as ED by the green curve) reveals the partial scattering cancellation with Fano-like spectral windows. Therefore, it can be concluded that the observed Fano-like resonances are closely attributed to the anapole excitation, i.e. the destructive interference between the electric and toroidal dipoles due to their identical radiation pattern. Furthermore, anapole states naturally lead to the formation of localized fields with strong enhancements^66^, which opens up new possibilities in spectroscopic and biochemical sensing and quantum nanophotonic applications^67^. Here, the observed scattering dips are referred as the first- and second-order anapole states^68–71^, labeled with $AM_1$ and $AM_2$ in Fig.2, respectively. To explore origin of the cancellation of P and TD moments observed in ED spectrum, considering dominant components in the direction of applied electric field (*x*-axis), the phases of Cartesian electric ${\phi _P}$ and toroidal dipole moments ${\phi _{TD}}$ and their differences $\Delta \phi = {\phi _P} - {\phi _{TD}}$ are shown in Fig. 2b. It is visible that in our spectral range of interest, the phase difference is very close to $\pi$ radiant, indicating that P and TD moments are almost out of phase. This fulfills the cancellation observed in Fig. 2a.

To further illustrate excitation of the so-called anapole modes, the surface charge density distributions on the particles at the position of characteristic dips are shown in Fig.2c,d, where the observation plane is considered in the mid-height of the structure. According to the dipolar moment orientation of the particles (dark arrows in figure), the $AM_1$ mode of our proposed oligomer shows dual displacement electric current loops within the structure. These moments are aligned head-to-tail and form a pair of out-of-plane counter-oriented magnetic dipoles, which corresponds to the superposition of a magnetic quadrupole moment and a head-to-tailed loop of magnetic dipole moments perpendicular to the meta-molecule surface. The latter dipolar configuration implies the excitation of a so-called dynamic toroidal dipole moment^69,72,73^ directed parallel to the *x*-axis. On the other hand, the $AM_2$ mode possesses two pairs of such poloidal current loops and result in four field zeroes along the y-direction, indicating a clear combination of the $AM_1$ mode and an accompanied standing wave character. This phenomenon can be explained by the formation of hybrid Mie-Fabry-Perot modes^74^ or the superposition of several internal modes^75^. Further, Fig.2e,f represents the spatial distributions of near-field amplitude $\left( {{{\left| \mathbf{E} \right| } \big / {\left| {\mathbf{E_{inc}}} \right| }}} \right)$ of the oligomer at two characteristic anapole wavelengths, where the near field enhancements provided by the hot spots of $AM_1$ and $AM_2$ modes are 44.7 and 91.5, respectively.

To go further into the aforementioned mode interaction and to elucidate the constitution influence, in what follows, we investigate the effect of the nanoparticles size as well as the illumination conditions on overall spectra. We expect to observe that changing the value of these parameters influences both the direct excitation as well as the mutual coupling between resonant modes.

**Figure 3:**
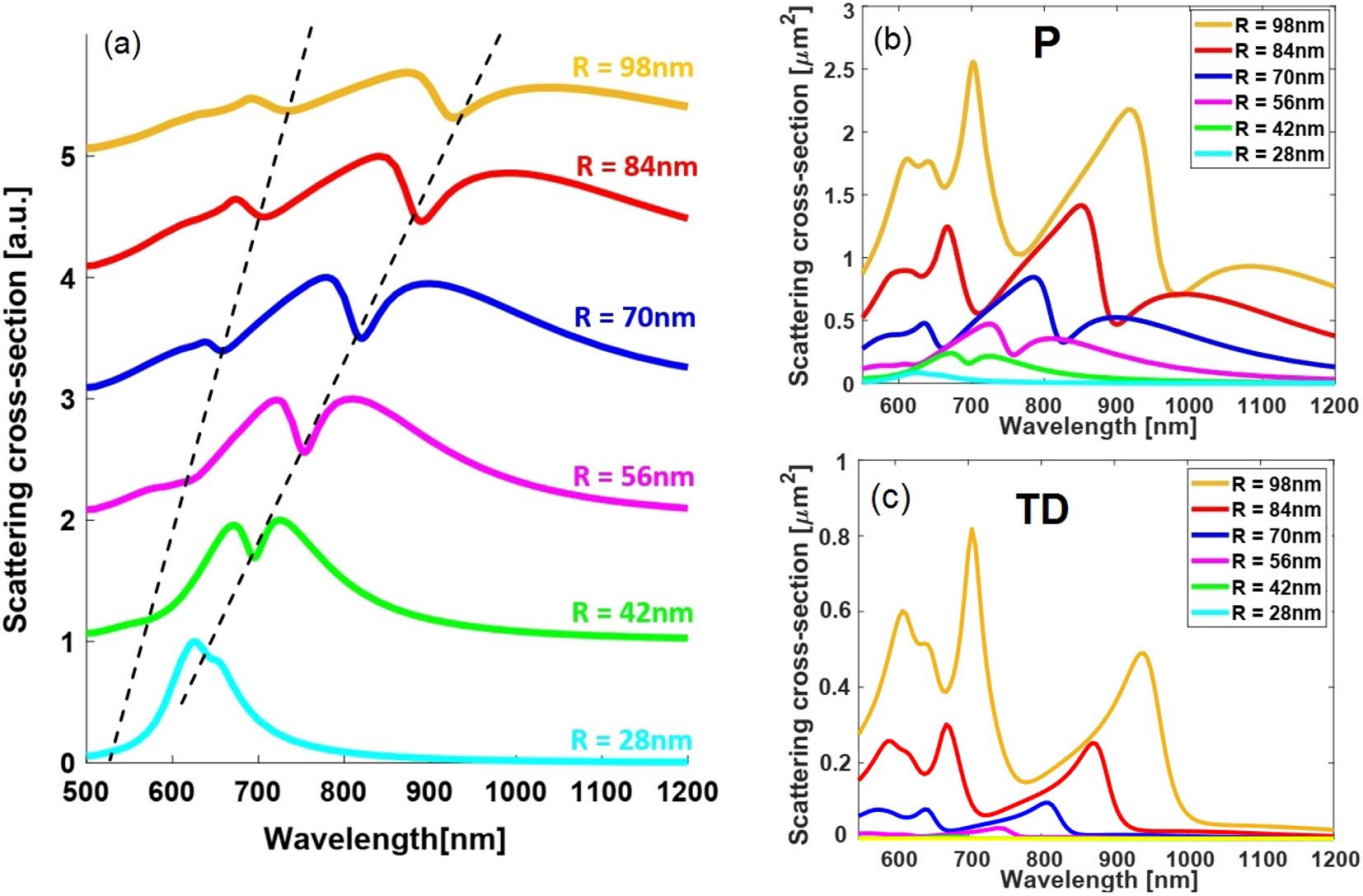
(**a**) Total SCS spectra of the oligomer structure illuminated by a normally-incident plane wave for different radii of particles, while the ratio between the radius and the gap between the particles being fixed. The remaining optical and geometrical parameters are the same as Fig.1. Contribution of the electric (P) and toroidal (TD) dipole multipoles are respectively illustrated in (**b**-**c**) to provide additional information regarding evolution of the Fano-like spectral line shapes.

#### Dependence of double-Fano resonance mode on the oligomer’s size

Here, we illustrate how the oligomer size affect evolution of the double-Fano spectral line shapes, a fact that critically determines the plasmonic structure design for SECARS applications. To this end, the size of oligomer under-investigation is scaled down by decreasing radius of the individual nanoparticles while keeping the radius to gap ratio fixed and equal to 7. The remaining optical and geometrical parameters are the same as Fig.1. From Fig.3a, it can be clearly observed that the Fano line profile with lower resonance wavelength ($AM_2$ state) gradually disappears with decreasing the radius *R* and the next one (representing $AM_1$ state) turns into a Lorentzian-shaped resonance band. Such transitions in the resonance line shapes can be quantitatively interpreted as follows:

The multipole expansion analysis starts from the induced polarization current density $\mathbf{ J }\left( \mathbf{ r } \right)$ in the nanoparticle, which given the convention ${e^{j\omega t}}$ for the harmonic electromagnetic fields, is calculated by^76^ $\mathbf{ J }\left( \mathbf{ r } \right) = j\omega \left( {{\varepsilon _p} - {\varepsilon _d}} \right) \mathbf{ E }\left( \mathbf{ r } \right)$ where $\omega$ is the frequency of the incident light in vacuum, $\mathbf{ E }\left( \mathbf{ r } \right)$ is the induced electric field, and ${\varepsilon _p} = {\varepsilon _0}{\varepsilon _{r,p}}$ and ${\varepsilon _d} = {\varepsilon _0}{\varepsilon _{r,d}}$ are the dielectric permittivities of the nanoparticles and background medium, respectively, ${\varepsilon _0}$ being the vacuum permittivity. The relative permittivities ${\varepsilon _{r,p}}$ and ${\varepsilon _{r,d}}$ are equal to the square of the corresponding refractive indices ${n_p},{n_d}$.

Let recall the recently obtained exact expression for the dipolar vector of electric field in the Cartesian coordinate basis^64^:

1 $$\begin{aligned} d_\alpha ^E = {1 \over {j\omega }}\left\{ {\int _V {{d^3}\mathbf{ r }{J_\alpha }{j_0}\left( {{k_d}r} \right) } + {{{k_d}^2} \over 2}\int _V {{d^3}\mathbf{ r }\left[ {3\left( {\mathbf{ r } \cdot \mathbf{ J }} \right) {r_\alpha } - {r^2}{J_\alpha }} \right] {{{j_2}\left( {{k_d}r} \right) } \over {{{\left( {{k_d}r} \right) }^2}}}} } \right\} \end{aligned}$$

where ${k_d} = \omega  / {{v_{d}}}$ and ${v_d} = {c  / {{n_d}}}$ are respectively the wave vector and speed of light in the surrounding environment, and $\mathbf{r} = r\hat{r}$ is the radius vector of a volume element inside the scattering medium (volume *V*). Making the small argument approximation to the spherical Bessel functions with terms up to fourth order ${\left( {{k_d}r} \right) ^4}$ (See Eq. (7) in “Methods”), and then grouping the contributions with the same power of $\left( {{k_d}r} \right)$, we obtain

2 $$\begin{aligned} d_\alpha ^E&\approx \underbrace{{1 \over {j\omega }}\int _V {{d^3}\mathbf{ r }{J_\alpha }} }_{\bar{l} = 0} + \underbrace{{1 \over {j\omega }}{{k_d^2} \over {10}}\int _V {{d^3}\mathbf{ r }\left[ {\left( {\mathbf{ r } \cdot \mathbf{ J }} \right) {r_\alpha } - 2{r^2}{J_\alpha }} \right] } }_{\bar{l} = 0, \bar{l} = 2} + \underbrace{{1 \over {j\omega }}{{k_d^4} \over {10}}{1 \over {28}}\int _V {{d^3}\mathbf{ r }{r^2}\left[ {3{r^2}{J_\alpha } - 2\left( {\mathbf{ r } \cdot \mathbf{ J }} \right) {r_\alpha }} \right] } }_{\bar{l} = 2} \nonumber \\&= {P_\alpha } - j{k_d}\left( {{T_\alpha } + {{k_d^2} \over {10}}\bar{R}_\alpha ^T} \right) \mathrm{{ }}\left( {\alpha = x,y,z} \right) \end{aligned}$$

where the contributions coming from $\bar{l}$-th order spherical Bessel function of the first kind $\left( {{j_{\bar{l}}}\left( \cdot \right) } \right)$ are indicated. Here, the ${\left( {{k_d}r} \right) ^0}$ term, ${P_\alpha }$ in Eq. (2), is the well-known small source approximation of the electric dipole moment of a current density distribution ${J_\alpha }$, and the ${\left( {{k_d}r} \right) ^2}$ term, ${T_\alpha }$ in Eq. (2), represents the so-called toroidal dipole moment. In addition, ${\left( {{k_d}r} \right) ^4}$ term shown by $\bar{R}_\alpha ^T$ in Eq. (2) is referred to as the mean-square radii of the toroidal dipole moment^77^. Notably, the ${T_\alpha }$ integral contains contributions from the two integrals in Eq. (1): It is the sum of the terms of order ${\left( {{k_d}r} \right) ^2}$ coming from both ${j_0}\left( {{k_d}r} \right)$ in the first integral of Eq. (1) and ${j_2}\left( {{k_d}r} \right)$ in the second one. By further decreasing the particle’s size parameter ${ {{k_d}r} }$ (e.g. having around 30 nm in radius disks), the highest order terms of ${j_0}\left( {{k_d}r} \right)$ become negligible compared to the first one, leading to exclusion of term corresponding to $\bar{l} = 0$ in ${T_\alpha }$ formula. Accordingly, the toroidal dipole expression tends to a negligible value and causes the mutual coupling required for the excitation of a non-radiating anapole state, namely $\mathbf{ P } = j{k_d}\mathbf{ T }$, to be destroyed. Following this discussion, Fig.3b,c clearly illustrate that the P multipolar contribution shows a transition to Lorentzian line profile (Fig.3b) and the TD becomes negligible (Fig.3c). This analysis not only gives insight regarding the design of SECARS substrate, but also serves as an additional verification making the anapole nature of the resultant scattering dips manifest.

**Figure 4:**
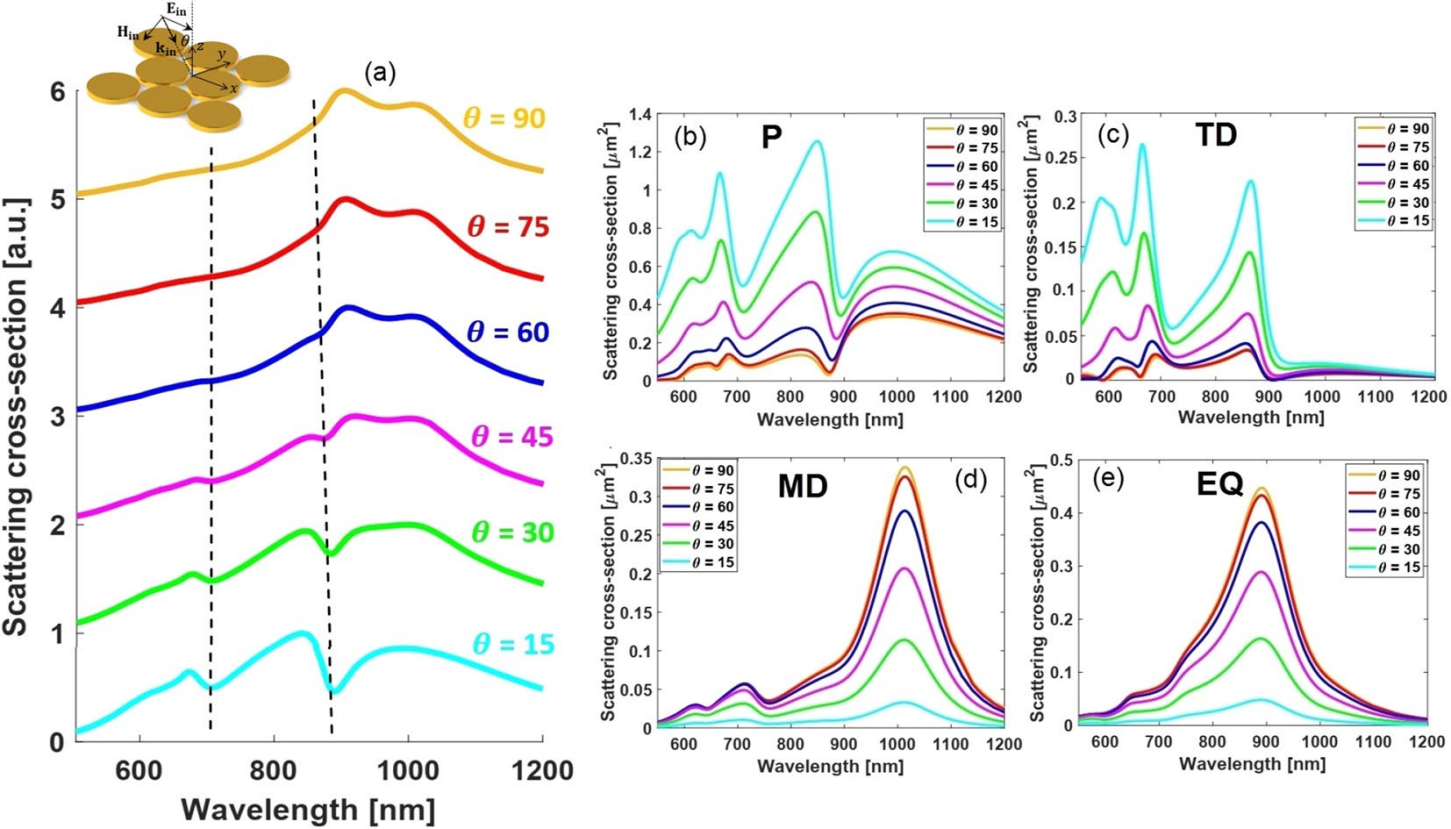
(**a**) Scattering spectra of the proposed oligomer ($R = 84\,{\text{nm}}$, $g = 12\,{\text{nm}}$, $h = 20\,{\text{nm}}$) as the incident angle increases from 0 to 90 with an increment of 15. Evolution of the (**b**) electric dipole P (**c**) toroidal dipole TD (**d**) magnetic dipole MD, and (**e**) electric quadrupole EQ modes with the incident direction is included to distinguish the dominant resonance in each incident angle.

#### Dependence of double-Fano resonance mode on the excitation angle

To further demonstrate the tailoring overall spectra characteristic, the angle of incident illumination is also varied from 0 to 90 and the spectra evolution is investigated. This study provides insights into how increasing excitation angle will affect the spectral response of our designed nanostructure. Considering the initial oligomer design (Fig.1), it is found that the observed anapole states progressively vanish from the total scattering spectra as the incident angle becomes more grazing (Fig.4a). The reason of this behavior can be clarified through identifying the multipolar contributions in terms of different incident angles, as shown in Fig.4b–e). It can be concluded that such evolution of scattering spectra is mainly a consequence of changes in the direct coupling between the incident plane wave and the different multipoles sustained by the structure. So, the amplitude of MD and EQ multipoles increases by the incident angle as a result of an increase of their interplay with the illuminated plane wave. On the other hand, P and TD multipoles amplitude is weakened and their spectral line profile noticeably changes with increasing the incident angle as well. These performances can be qualitatively interpreted as a consequence of a change in their direct coupling with incident plane wave combined with changes in their mutual interactions, respectively. It is pointed out that spectrum evolution of the MQ is close to that of TD given the existed correlation between these two multipoles along with an electric octopule moment^54^.

**Figure 5:**
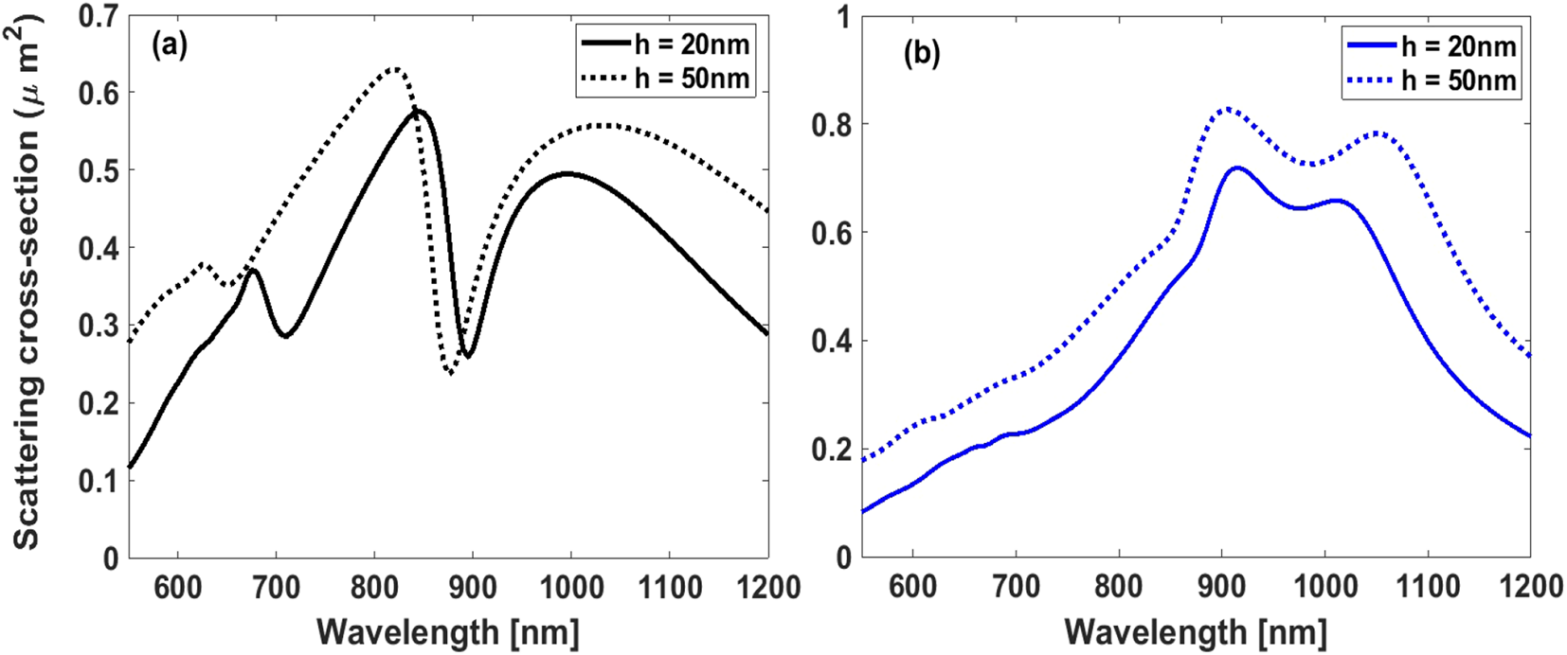
Total SCS of the proposed oligomer for different heights of gold nano-disks (**a**) normal incidence, and (**b**) oblique incidence of angle 60. The remaining parameters are the same as those in Fig.2.

In order to accomplish the goal of our study, one may take into account dependence of the dielectric function of gold nano-disks which can be affected by surface scattering of nano-scale metallic particles. The surface scattering can modify dispersion of gold nanoparticles which are much smaller than the incoming wavelength. As the thickness of the metal increases, discrepancy between the dielectric function of bulk and nano-scale materials tends to be negligible^78,79^. For all of the results discussed above, we disregarded modification of gold nano-disks dielectric function affected by surface scattering mechanism. To carry out validation of our approach, it should be noticed that our proposed oligomer is illuminated through an s-polarized plane wave whose electric field is parallel to the surface of nano-disks having notably large diameters (168 nm in diameter). Therefore, excitation of free electrons will be dominant along the transverse direction and, as a result, height of the nano-disks will not considerably affect scattering performance of our plasmonic structure. To clearly illustrate, in Fig.5 the scattering cross section for different heights of nano-disks are calculated in both normal and oblique incidence $\left( {\theta = {{60}^ \circ }} \right)$. It can be observed that by increasing the thickness of gold nano-disks, apart from a slightly higher scattered signal of the thicker disks together with minor spectral shifts of the modal resonances potentially used in SECARS, no significant changes are experienced in the total scattering spectra.

### Spectral manipulation of anapole states for SECARS applications

In this section, we demonstrate that the optical response of our proposed plasmonic oligomer can be manipulated such that the incident beams at the pump $\omega _p$ and Stokes $\omega _s$ frequencies interact coherently with medium to generate the emission of the CARS photons at the blue-shifted anti-Stokes frequency $\omega _{As}$, i.e., ${\omega _{As}} = 2{\omega _s} - {\omega _p}$^51^. Note that in the CARS process, one of a four-wave mixing processes involving three laser fields interacting with the sample, not only the frequencies of the incident and scattered light, but also the wavevectors of each beam including phases should be considered. This fact demonstrates to be critical in the first experimental implementation into microscopy using noncollinear excitation of pump and Stokes visible dye laser beams^80^. In the plane-wave approximation for a non-absorbing material, the CARS signal intensity depends linearly on the term ${\text{sinc}}^{2} \left( {\Delta \vec{k} \cdot \vec{l}/2} \right)$, where *l* is the thickness of sample, $\Delta {\vec {k}}$ the phase mismatch $\Delta \vec{k} = {{\vec {k}}_p} - 2{{\vec {k}}_s} + {{\vec {k}}_{As}}$ and ${\vec{k_i}}\left( {i = p,s,As} \right)$ are the wavevectors for each one of the interacting (pump, Stokes and anti-Stokes) lights. As a consequence, generation of the CARS signal needs the phase-matching condition $\Delta \vec{k} \cdot \vec{l} \ll \pi$ to be fulfilled, which reveals the coherent nature of the CARS process. Furthermore, for a very thin film the phase-matching condition is usually satisfied both in forward and backward directions relative to the propagation direction of the excitation beams^81^. When using SECARS substrates, the effective thickness where light interaction is enhanced typically lies under a subwavelength scale, as it occurs in our proposal, thus enabling the phase-matching condition to be fully satisfied. Nevertheless, such condition is further constrained to excitation under normal incidence when using a coplanar array of metallic octamer structures^82^.

The observed non-radiating anapole states are interesting for CARS applications due to the several reasons: (1)Given the lack of scattering and radiation loss in a dipole channel, a highly concentrated electromagnetic near-field in the center of the structure is generated boosting nonlinear effects, (2)The higher-order anapole states provide stronger energy localization and narrower resonance windows, an exotic feature that is advantageous for nonlinear higher-harmonic generators, (3)The anapole wavelengths can be easily measured through identifying the far-field scattered spectrum of white light from the structure. As a consequence, our observed anapole states are nearly ideally suited for the SECARS process.

**Figure 6:**
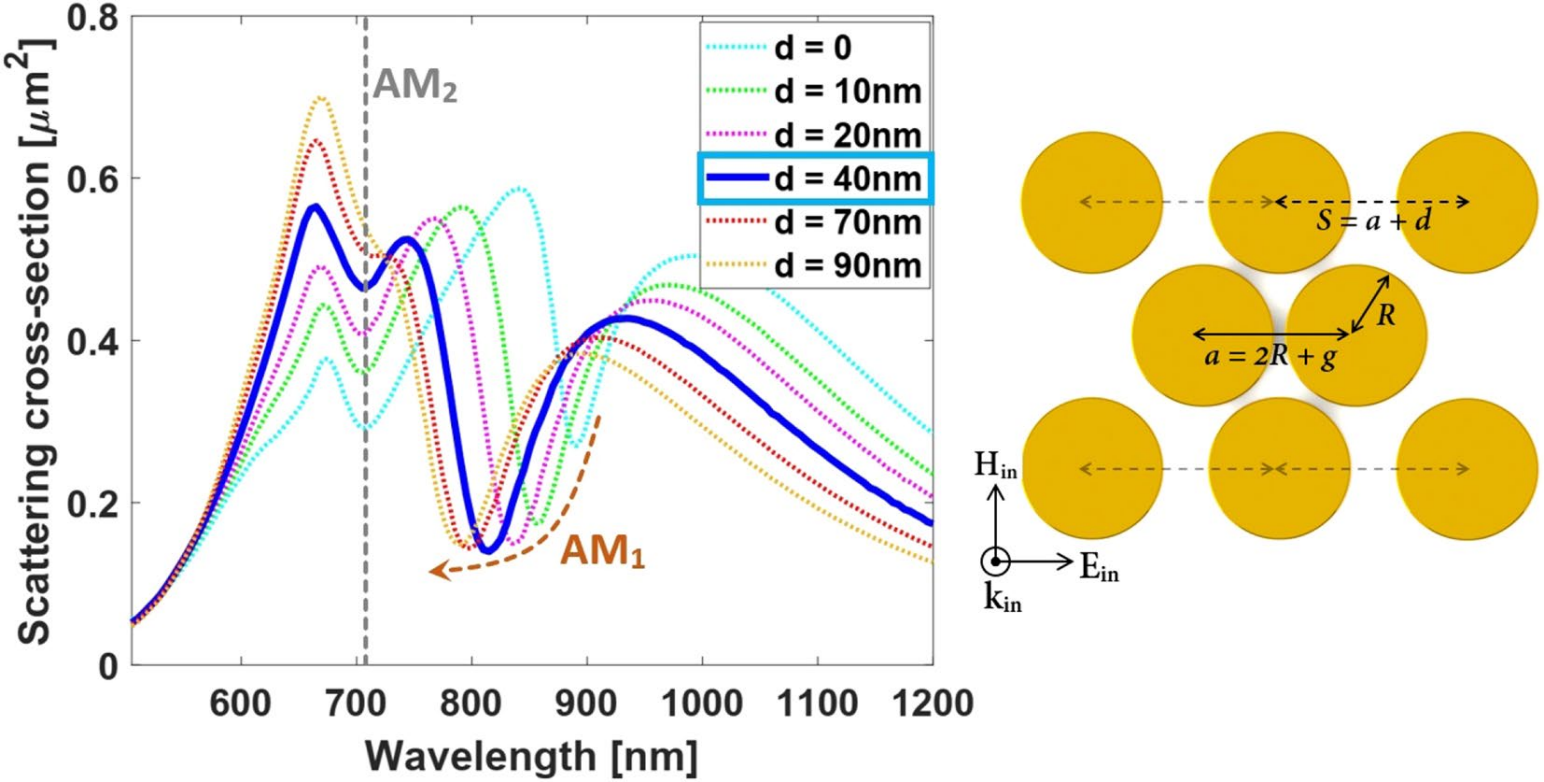
Simulated total SCS of our proposed oligomer considering various gap distances for corner particles denoted by the deviation $S = a + d$. The other geometrical parameters are chosen as the same as those in Fig.1. The results show the independent spectral tunability of the first-order anapole mode ($AM_1$ mode) from the second one. It can be seen that the SECARS condition is fulfilled for the deviation value of $d = 40\,{\text{nm}}$.

Revisiting the surface charge density distributions corresponding to $AM_1$ and $AM_2$ anapole modes shows that the corner particles of the oligomer under study only contribute to the formation of external current loops (not the central ones) in case of $AM_2$ mode whereas these particles are essential elements to produce current loops corresponding to $AM_1$ mode. Since electric field hot spots of two anapole states are mostly concentrated in the gap region of the central dimmer as seen in Fig.2d–e), it can be concluded that the central current loops play the main role in characteristics of the observed Fano-like line profiles. It has been shown previously that the depth of Fano-like spectral windows can be modulated by varying the LSPR coupling strength between adjacent nanoparticles. Furthermore, the spectral position of scattering dips can be tuned through controlling the radius of associated current loops^83,84^. Following this idea, it is expected that modulating the gap distance of the corner particles here will change the coupling strength between the nanoparticles as well as the size of main current loops in case of $AM_1$ mode and the size of external current loops in case of $AM_2$ anapole mode. Therefore, adjusting the gap separation between corner particles will result variations of both resonance depth and spectral position for the $AM_1$ mode. Concerning the $AM_2$ mode, on the other hand, such an adjustment will only affect depth of the scattering dip without displacing its spectral position. The reason of this performance can be inferred from the size of the main (internal) current loops of the $AM_2$ mode remaining unchanged. To examine the above qualitative analysis, we vary the gap distances of the corner particles on the top and bottom sides by considering different values of deviations *d* from the initial value $g = 12\,{\text{nm}}$. We numerically calculate the total scattering spectra of the resultant system. The simulated responses are shown in Fig.6 where $S = 2R + g + d$, demonstrating the agreement with the aforementioned analysis of the anapole modes.

Interestingly, the obtained results exhibit independent spectral tunability of the first-order anapole state ($AM_1$ mode) with respect to the variations of gap distance of the corner particles on the top and bottom sides. This feature is the design key to simply adjust spectral response of the first-order anapole mode independent from the second one for SECARS applications.

**Figure 7:**
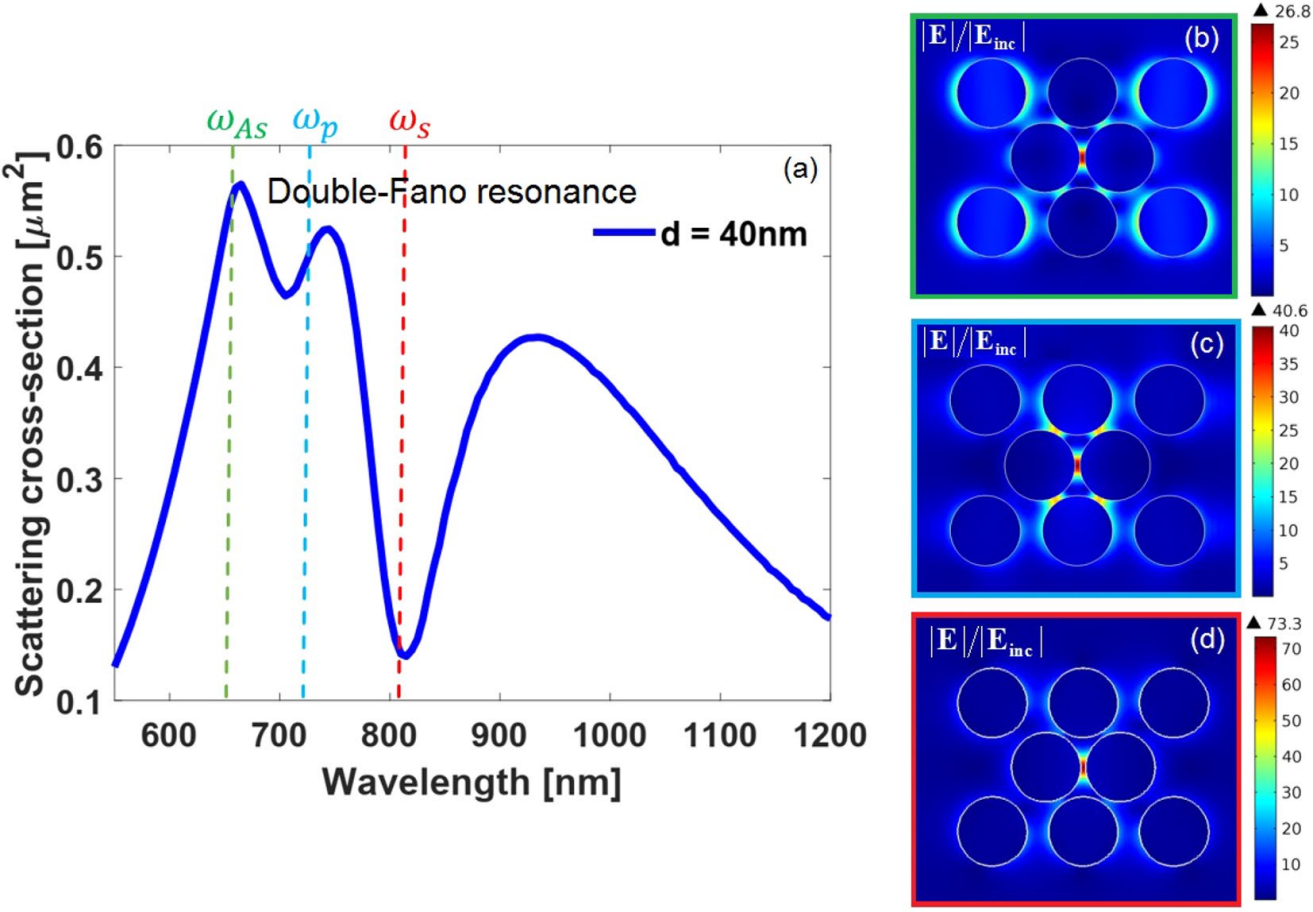
(**a**) Simulated total SCS spectra of our designed SECARS substrate with deviation value of $d = 40\,{\text{nm}}$ where the vertical dashed green, blue, and red lines respectively denote the ${\lambda _{As} = 653\,{\text{nm}}}$, ${\lambda _{p} = 720\,{\text{nm}}}$, and ${\lambda _{s} = 803\,{\text{nm}}}$ satisfying the SECARS condition as ${\omega _{As} = 2\omega _p - \omega _s }$. Spatial distributions of the electric field intensity corresponding to the three involved (**b**) anti-Stokes, (**c**) pump, and (**d**) Stokes signals, when monitored at the middle height of the oligomer $\left( {z = 10\,{\text{nm}}} \right)$.

It should be most advantageous for SECARS enhancements to tune the two anapole states at the pump and Stokes wavelengths while the superradiant shoulder (at the blue side of the both anapole states) at the CARS wavelength. This strategy results minimizing the radiation losses at both excitation wavelengths while maximizing the far-field coupling at the wavelength of anti-Stokes emission^85^. The reduction of the scattered light at the wavelengths associated with the anapole states ensures efficient energy coupling into the nanostructure, thus generating highly localized, intense field enhancements. In addition, the highly-radiative shoulder provides an enhanced propagation of CARS signal to the far-field region. It can be seen the SECARS condition is fulfilled for the deviation value of $d = 40\,{\text{nm}}$ as shown in Fig.7. The corresponding wavelengths are ${\lambda _{As}} = 653\,{\text{nm}}$, ${\lambda _{p}} = 720\,{\text{nm}}$, ${\lambda _{s}} = 803\,{\text{nm}}$, where the Stokes frequency and the pump laser excitation frequency matches to the first- and second-order anapole states respectively and the high-frequency shoulder overlaps with the anti-Stokes mode. These three involved frequencies in scattering spectrum obviously satisfy ${\omega _{As}} = 2{\omega _s} - {\omega _p}$.

Efficiency of the SECARS substrate is identified by maximizing the total electromagnetic field EF at the anti-Stokes frequency through the relation^86^:

3 $$\begin{aligned} {G_{SECARS}} = {\left| {{{E\left( {{\omega _p}} \right) } \bigg / {{E_0}\left( {{\omega _p}} \right) }}} \right| ^4} \times {\left| {{{E\left( {{\omega _s}} \right) } \bigg / {{E_0}\left( {{\omega _s}} \right) }}} \right| ^2} \times {\left| {{{E\left( {{\omega _{As}}} \right) } \bigg / {{E_0}\left( {{\omega _{As}}} \right) }}} \right| ^2} = g_p^4 \times g_s^2 \times g_{As}^2 \end{aligned}$$

where $g_p$, $g_s$, and $g_{As}$ are the localized electric field enhancements corresponding to the pump, Stokes, and anti-Stokes frequencies, respectively. Thus, an appropriate plasmonic substrate staisfying Raman dipole-forbidden band distribution condition of the molecule will present strong localized hot spots depending on three characteristic frequencies. Spatial overlap of the hot spots at three resonant modes that form the “mixed frequency coherent mode” is important for SECARS application and it will be useful to reach a strong EF. The spatial electric field distributions at three involved wavelengths of our designed substrate are shown in the Fig.7b–d. The fields are monitored at the middle height of the oligomer $\left( {z = 10 \,{\text{nm}}} \right)$ and they confirm high simultaneous electric field enhancement with the hot spots at the same spatial locations (gap of the central dimer), both necessary conditions to yield an efficient SECARS substrate. The corresponding electric field EFs are $g_s = 73.3$, $g_p = 40.6$, and $g_{As} = 26.7$ leading to the total EF of $G_{SECARS} = 1.05 \times {10^{13}}$ .

Through adjusting geometrical parameters of our proposed oligomer, the wavelengths of anti-Stokes, pump, and Stokes modes can be freely tuned, providing a set of different wavelengths to be used in SECARS applications. In Table 1, we have summarized the value of SECARS EF for different radius of nanoparticles where the radius to gap ratio is considered to be fixed $(R/g = 7)$. For the design parameters of $R = 77\,{\text{nm}}$ and $g = 11\,{\text{nm}}$, anti-Stokes, pump and Stokes wavelengths are obtained as ${\lambda _{As}} = 634\,{\text{nm}}$, ${\lambda _{p}} = 695\,{\text{nm}}$, ${\lambda _{s}} = 769\,{\text{nm}}$, respectively. Here, the pump frequency falls into the visible region which is of great interest in most SECARS applications. Using this design, one can utilize a short wavelength laser to pump the substrate and materials. In addition, it should be noted that gap spacing between nanoparticles plays a crucial role in the resonance modes and hot spot field enhancement. Shrinking the gap distance to $g = 8\,{\text{nm}}$ while the remaining parameters being the same as those in Fig. 2 yields a significant SECARS enhancement ${G_{SECARS}} = {57^2} \times {68.5^4} \times {118^2} \approx {10^{15}}$ (see Figs. S2 and S3 in Supplementary Information) which is almost two orders of magnitude larger than those having the same gap size reported in the literature^52^, along with a red-shift of the characteristics wavelengths.

**Table 1:** Evaluation of the SECARS EF with the different configuration parameters where radius to gap ratio is considered to be constant and equal to 7.

Structure	$R \left( {\,{\text{nm}}} \right)$	$g \left( {\,{\text{nm}}} \right)$	$d \left( {\,{\text{nm}}} \right)$	Anti-stokes		Pump		Stokes		${G _{SECARS}}$
				$$\lambda \left( {\,{\text{nm}}} \right) $$	$${g _{as}}$$	$$\lambda \left( {\,{\text{nm}}} \right) $$	$$ {g _{p}}$$	$$\lambda \left( {\,{\text{nm}}} \right) $$	$$ {g _{s}}$$	
1	77	11	40	634	23.5	695	43.3	769	80.7	$1.3 \times {10^{13}}$
2	84	12	40	653	26.7	720	40.6	803	73.3	$1.05 \times {10^{13}}$
3	91	13	40	668	25.5	742	38.2	835	68.1	$6.4 \times {10^{12}}$

Let us conclude our discussion by highlighting that our proposal might potentially optimize its performance through a fullwave Maxwell inverse design^87^. For instance, tapered ultranarrow gaps enable field enhancements exceeding ${10^{2}}$ in Ref.^88^, suggesting that surface engineering of the central dimer in our nanostructure thus shrinking the interparticle gap domain while maintaining the gap distance fixed might be favorable for the generation of higher CARS signals. In addition, by optimizing the oligomer’s geometrical parameters, we can tune the plasmonic substrate geometry so that its multiple resonances matches to the frequencies of the pump, Stokes, and anti-Stokes lights in the visible as well as near infrared spectral range.

## Conclusion

In summary, we have demonstrated an engineered assembly of metallic disk-shaped nanoparticles to be a highly-efficient free-standing substrate for SECARS application. To this end, we first propose a simple plasmonic oligomer structure supporting double-Fano resonance line shapes, which serves as a basis for our proposed SECARS substrate. Expanding the total field scattered by the whole structure into Cartesian multipolar basis reveals that the two observed characteristic dips corresponds to the so-called non-trivial first- and second-order anapole states. Distribution of electric current loops associated with these anapole modes suggests independent spectral tunability of the first-order anapole state through modulating gap distance of the corner particles. This feature along with giant electric field enhancements provided by anapole states offers a simple design procedure in the context of SECARS applications. Notably, the electric field “hot spots” corresponding to three involved wavelengths, i.e., anti- Stokes, pump, and Stokes, are localized at the same spatial position, which can be exploited to achieve a huge CARS signal field enhancement. The estimated total enhancement factor of SECARS process for our proposed design is up to ${10^{15}}$ providing the highest enhancement ever-obtained for the inter-disk spacing of $g = 8\,{\text{nm}}$.

## Methods

### Multipole expansion method

In standard electrodynamic textbooks^89,90^, the spherical multipoles result from the multipolar decomposition of electromagnetic fields in terms of the spherical vector harmonics. However, the complexity in interpreting the spherical basis is caused the spherical multipoles not to be analyzed directly. To improve this situation, recently exact expressions for the localized charge current density multipoles in the Cartesian basis were developed^64,91^. It is noted that the obtained expression set implicitly comprises toroidal multipole moments.

#### Exact Cartesian multipole decomposition

In the Cartesian coordinate system with the origin coinciding with the mass center of the nanoparticle, the ordinary ED moment of the nanoparticle is expressed by Eq. (1) in the main text, and the other leading multipoles up to MQ are described as

4 $$\begin{aligned} d_\alpha ^M&= {3 \over 2}\int _V {{d^3}\mathbf{ r }{{\left( {\mathbf{ r }} \times \mathbf{ J } \right) }_\alpha }{{{j_1}\left( {{k_d}r} \right) } \over {{k_d}r}}} \end{aligned}$$

5 $$\begin{aligned} Q_{\alpha \beta }^E&= {3 \over {j\omega }}\int _V {{d^3}\mathbf{ r }\left\{ {\left[ {{r_\alpha }{J_\beta } + {r_\beta }{J_\alpha } - {2 \over 3}{\delta _{\alpha \beta }}\left( {\mathbf{ r } \cdot \mathbf{ J }} \right) } \right] {{{j_1}\left( {{k_d}r} \right) } \over {{k_d}r}}} \right. } \nonumber \\&\quad \left. { + 2{k^2}\left[ {5\left( {\mathbf{ r } \cdot \mathbf{ J }} \right) {r_\alpha }{r_\beta } - {r^2}\left( {{r_\alpha }{J_\beta } + {r_\beta }{J_\alpha }} \right) - {r^2}{\delta _{\alpha \beta }}\left( {\mathbf{ r } \cdot \mathbf{ J }} \right) } \right] {{{j_3}\left( {{k_d}r} \right) } \over {{{\left( {{k_d}r} \right) }^3}}}} \right\} \end{aligned}$$

6 $$\begin{aligned} Q_{\alpha \beta }^M&= 5\int _V {{d^3}\mathbf{ r }\left[ {{r_\alpha }{{\left( {\mathbf{ r } \times \mathbf{ J }} \right) }_\beta } + {r_\beta }{{\left( {\mathbf{ r } \times \mathbf{ J }} \right) }_\alpha }} \right] {{{j_2}\left( {{k_d}r} \right) } \over {{{\left( {{k_d}r} \right) }^2}}}} \end{aligned}$$

where $d_\alpha ^M$, $Q_{\alpha \beta }^E$, $Q_{\alpha \beta }^M$ are respectively the magnetic dipole (MD), electric quadrupole (EQ), and magnetic quadrupole (MQ) tensors, $\delta$ is the Dirac delta function, and subscripts $\alpha ,\beta = x,y,z$. Commonly, a long-wavelength approximation is utilized making the above expressions sufficiently straightforward. The so-called toroidal dipole moments are derived explicitly by this kind of formulation.

#### Approximate Cartesian multipole decomposition

Having made the small argument approximation to the spherical Bessel functions in exact Cartesian multipole equations and keeping terms up to fourth order ${\left( {{k_d}r} \right) ^4}$ as

7 $$\begin{aligned} {j_0}\left( {{k_d}r} \right)&\approx 1 - {{{{\left( {{k_d}r} \right) }^2}} \over 6} + {{{{\left( {{k_d}r} \right) }^4}} \over {120}} \nonumber \\ {j_1}\left( {{k_d}r} \right)&\approx {{\left( {{k_d}r} \right) } \over 3} - {{{{\left( {{k_d}r} \right) }^3}} \over {30}} \nonumber \\ {j_2}\left( {{k_d}r} \right)&\approx {{{{\left( {{k_d}r} \right) }^2}} \over {15}} - {{{{\left( {{k_d}r} \right) }^4}} \over {210}} \nonumber \\ {j_3}\left( {{k_d}r} \right)&\approx {{{{\left( {{k_d}r} \right) }^3}} \over {105}} \end{aligned}$$

the top leading multipolar moments for the meta-molecule assembly are obtained as

8 $$\begin{aligned} d_\alpha ^M&\approx \underbrace{{1 \over 2}\int _V {{d^3}\mathbf{ r }{{\left( {\mathbf{ \mathbf{r} } \times \mathbf{ J }} \right) }_\alpha }} }_{d_\alpha ^{M,Car}} - {{k_d^2} \over {10}}\underbrace{{1 \over 2}\int _V {{d^3}\mathbf{ r }{r^2}{{\left( {\mathbf{ r } \times \mathbf{ J }} \right) }_\alpha }} }_{\bar{R}_m^2} = d_\alpha ^{M,Car} - {{k_d^2} \over {10}}\bar{R}_m^2 \end{aligned}$$

9 $$\begin{aligned} Q_{\alpha \beta }^E&\approx 6\left\{ {\underbrace{{1 \over {j2\omega }}\int _V {{d^3}\mathbf{ r }\left[ {{r_\alpha }{J_\beta } + {r_\beta }{J_\alpha } - {2 \over 3}{\delta _{\alpha \beta }}\left( {\mathbf{ r } \cdot \mathbf{ J }} \right) } \right] } }_{Q_{\alpha \beta }^{E,Car}}} \right. \nonumber \\&\quad \left. { - j{{{k_d}} \over 3}\underbrace{{1 \over {28{v_d}}}\int _V {{d^3}\mathbf{ r }\left[ {4{r_\alpha }{r_\beta }\left( {\mathbf{ r } \cdot \mathbf{ J }} \right) - 5{r^2}\left( {{r_\alpha }{J_\beta } + {r_\beta }{J_\alpha }} \right) + 2{r^2}\left( {\mathbf{ r } \cdot \mathbf{ J }} \right) {\delta _{\alpha \beta }}} \right] } }_{Q_{\alpha \beta }^T}} \right\} \nonumber \\&= 6\left\{ {Q_{\alpha \beta }^{E,Car} - j{{{k_d}} \over 3}Q_{\alpha \beta }^T} \right\} \end{aligned}$$

10 $$\begin{aligned} Q_{\alpha \beta }^M&\approx \underbrace{{1 \over 3}\int _V {{d^3}\mathbf{ r }\left[ {{r_\alpha }{{\left( {\mathbf{ r } \times \mathbf{ J }} \right) }_\beta } + {r_\beta }{{\left( {\mathbf{ r } \times \mathbf{ J }} \right) }_\alpha }} \right] } }_{Q_{\alpha \beta }^{M,Car}} - k_d^2\underbrace{{1 \over {42}}\int _V {{d^3}\mathbf{ r }{r^2}\left[ {{r_\alpha }{{\left( {\mathbf{ r } \times \mathbf{ J }} \right) }_\beta } + {r_\beta }{{\left( {\mathbf{ r } \times \mathbf{ J }} \right) }_\alpha }} \right] } }_{\bar{R}_{\alpha \beta }^{2,{Q_m}}} \nonumber \\&= Q_{\alpha \beta }^{M,Car} - k_d^2\bar{R}_{\alpha \beta }^{2,{Q_m}} \end{aligned}$$

where $d_\alpha ^{M,Car}$, $Q_{\alpha \beta }^{E,Car}$, $Q_{\alpha \beta }^{M,Car}$, and $Q_{\alpha \beta }^T$ denote respectively magnetic dipole, electric quadrupole, magnetic quadrupole, and toroidal quadrupole moments of Cartesian multipole decomposition. In addition, $\bar{R}_m^2$ and $\bar{R}_{\alpha \beta }^{2,{Q_m}}$ represent the mean-square radii corrections for the magnetic dipole and magnetic quadrupole moments, respectively^77^.

The partial scattering cross-sections corresponding to each multipole moment are given by

11 $$\begin{aligned} C_{sca}^{ED}&= {{{k^4}} \over {6\pi \varepsilon _0^2{{\left| {{E_{inc}}} \right| }^2}}}\sum \limits _\alpha {{{\left| {d_\alpha ^E} \right| }^2}} \end{aligned}$$

12 $$\begin{aligned} C_{sca}^{MD}&= {{{k^4}} \over {6\pi \varepsilon _0^2{{\left| {{E_{inc}}} \right| }^2}}}\sum \limits _\alpha {{{{{\left| {d_\alpha ^M} \right| }^2}} \over {v_d^2}}} \end{aligned}$$

13 $$\begin{aligned} C_{sca}^{EQ}&= {{{k^4}} \over {720\pi \varepsilon _0^2{{\left| {{E_{inc}}} \right| }^2}}}\sum \limits _{\alpha ,\beta } {{{\left| {{k_d}Q_{\alpha \beta }^E} \right| }^2}} \end{aligned}$$

14 $$\begin{aligned} C_{sca}^{MQ}&= {{{k^4}} \over {720\pi \varepsilon _0^2{{\left| {{E_{inc}}} \right| }^2}}}\sum \limits _{\alpha ,\beta } {{{{{\left| {{k_d}Q_{\alpha \beta }^M} \right| }^2}} \over {v_d^2}}} \end{aligned}$$

assuming $\left| {{\mathbf{ E }_{\mathrm{{in}}}}} \right|$ as the electric field amplitude of the incident plane wave. It is convenient to define also the magnitudes $C_{sca}^P$ and $C_{sca}^T$ as

15 $$\begin{aligned} C_{sca}^P&= {{{k^4}} \over {6\pi \varepsilon _0^2{{\left| {{E_{inc}}} \right| }^2}}}\sum \limits _\alpha {{{\left| {{P_\alpha }} \right| }^2}} \end{aligned}$$

16 $$\begin{aligned} C_{sca}^T&= {{{k^4}} \over {6\pi \varepsilon _0^2{{\left| {{E_{inc}}} \right| }^2}}}\sum \limits _\alpha {{{\left| {{T_\alpha } + {{k_d^2} \over {10}}\bar{R}_\alpha ^T} \right| }^2}} \end{aligned}$$

which correspond to effective cross-sections of the Cartesian electric and toroidal dipoles, respectively. These two dipoles may interfere constructively or destructively, depending on their relative phase difference, and their combined contribution to the electric dipole scattering cross-section is described in Eq. (11).

Considering the other higher order terms are neglected in the investigated nanoantenna, the total scattering cross-section $C_{sca}^{total}$ from the above multipoles can be approximated as

17 $$\begin{aligned} C_{sca}^{total} \approx C_{sca}^{ED} + C_{sca}^{MD} + C_{sca}^{EQ} + C_{sca}^{MQ} \end{aligned}$$

It is well worth mentioning that in spite of popularity of the approximate Cartesian multipoles (known as Cartesian multipole decomposition elements), they cannot exactly reconstruct the electrodynamic radiation fields and the related scattering phenomena in case of large particles as well as high refractive-index particles.

### Computational methods

Numerical simulations of the structure discussed in this study were carried out using the finite element method (FEM) with the implement of COMSOL Multiphysics, where Perfectly Matched layers (PML) were utilized to avoid spurious reflections at the surrounding boundaries of plasmonic nano-systems. The induced electric field **E(r)** inside the structure was calculated in the framework of the scattered-field formulation. To directly determine the total scattering cross section, the scattered flux in a closed surface surrounding the oligomer was calculated using the Poynting theorem. The surface charge density distributions are obtained by considering the skin effect and applying Gauss’ law during FEM calculations^92^. In all cases, a linearly-polarized light with the electric field polarization along the gap axis of the central dimer (*x*-direction) was used as the excitation source, where the propagation direction coincides with the *z*-axis. Here, ${\mathbf{ k }_{\mathbf{ in }}}$, ${\mathbf{ E }_{\mathbf{ in }}}$, and ${\mathbf{ H }_{\mathbf{ in }}}$ are the wave vector, electric field, and magnetic field of the incident light, respectively. To reach sufficiently high simulation accuracy the particles need to be finely meshed. Here the maximum mesh size is 8 nm. The parameter sweep module in this study is utilized for the spectrum simulation covering a range from 550 nm to 1200 nm.

## Supplementary Information

Supplementary Information.
