# Supplementary material for: Anapole-assisted giant electric field enhancement for surface-enhanced coherent anti-Stokes Raman spectroscopy
Source: Sci Rep. 2021 May 20;11:10639. doi: 10.1038/s41598-021-90061-5 (PMC8137709; doi:10.1038/s41598-021-90061-5)
Supplement: Supplementary file 1 — Supplementary Information. [file 41598_2021_90061_MOESM1_ESM.docx]

Supplementary Information

**Anapole-assisted giant electric field enhancement for surface-enhanced coherent anti-Stokes Raman spectroscopy**

Maryam Ghahremani^1,*^, Mojtaba Karimi Habil^2^, and Carlos J. Zapata-Rodriguez^3^

^1^ Photonics Research Laboratory, Center of Excellence for Applied Electromagnetic Systems, University of Tehran, North Kargar Ave., Tehran, Iran

^2^ Faculty of Physics, University of Tabriz, 51664 Tabriz, Iran

^3^ Department of Optics and Optometry and Vision Science, University of Valencia, 46100 Burjassot, Spain

Correspondence and requests for materials should be addressed to M.G. (ghahremani.maryam@ut.ac.ir)

Here, we aim to provide further insight into the discrepancy between directly calculated scattering cross section and the one obtained by Cartesian multipole summation, shown in Fig. 2 (a) in the main text. For this purpose, the exact multipole expansion method in spherical coordinate [1] is employed to determine scattering spectra for the first four modes of the design presented in Fig. 2. Simulation results are shown in Fig. S1, where the labels ED, MD, EQ, MQ represent electric dipole, magnetic dipole, electric quadrupole, and magnetic quadrupole, respectively. “Total Scat” is calculated from integrating energy flux via direct numerical simulation while “Sum Sph” is the summation of the first four modes. The two plots are in good accordance throughout most parts, except for the region with shorter wavelength, where higher-order multipoles should be taken into consideration. For the sake of comparison, the scattering cross-section obtained by the summation of Cartesian moments is shown in Fig. S1 (dotted-red line). It can be seen the shorter wavelength, the higher discrepancy coming from the long-wavelength approximations [2] used in Cartesian expansion method.


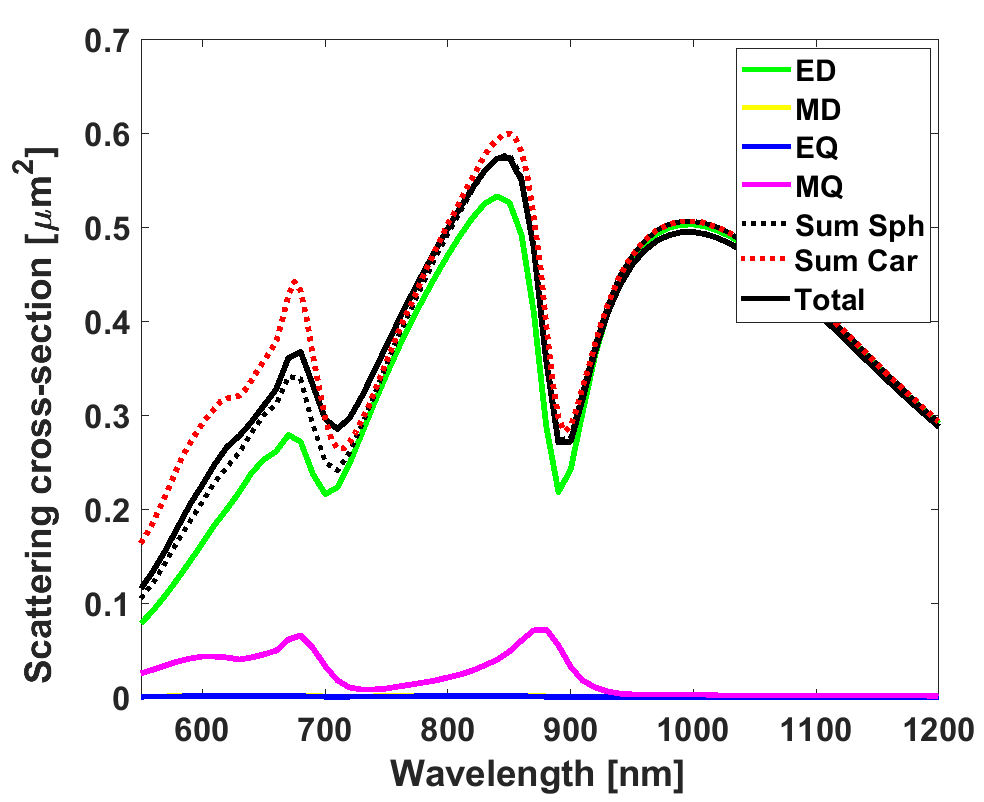


Fig. S1. Spectra of the scattering cross section and corresponding multipoles contributions calculated for the design presented in Fig. 2. “Sum Sph” and “Sum Car” are the summation of spherical (exact) and approximate Cartesian multipole contributions, respectively. “Total” is the scattering cross section obtained via direct numerical simulation; other abbreviations can be found in the text.

In what follows, we provide numerical simulations of our proposed plasmonic disk-based oligomer design analyzing its performance for the decreased inter-disk gap distance of $g=8nm$. Here nonlocal effects can be disregarded [3]. The remaining geometrical and structural parameters are the same as the design in Fig. 2$\left( R=84nm,h=20nm \right)$.

Fig. S2 represents the simulated total scattering spectrum of the structure for different deviation of corner particles. We show that the first order anapole mode (AM_1_) can be adjusted independently simply by tuning gap distance between corner particles. It is found that for the deviation value of $d=40nm$, two Fano minima and the super-radiant shoulder could just overlap to the wavelengths of the pumping (blue, 745 nm), Stokes (red, 822 nm), and anti-Stokes (blue, 682 nm) beams in SECARS technique (See Fig. S3 (a)). The spatial distribution of near-field amplitude $\left( \left| \mathbf{E} \right|/\left| \mathbf{E}_{\mathbf{0}} \right| \right)$ of the oligomer at three involved wavelengths are shown in Fig. S3 (b-d). The magnitude of SECARS EF for the proposed design is obtained as$G_{SECARS}={57}^{2}\times{68.5}^{2}\times{119}^{2}\approx{10}^{15}$. The strong **E**-field “hot spots” due to the excitation of first- and second- order anapole modes along with their same spatial localization offers our design as an efficient substrate for single-molecule detection.


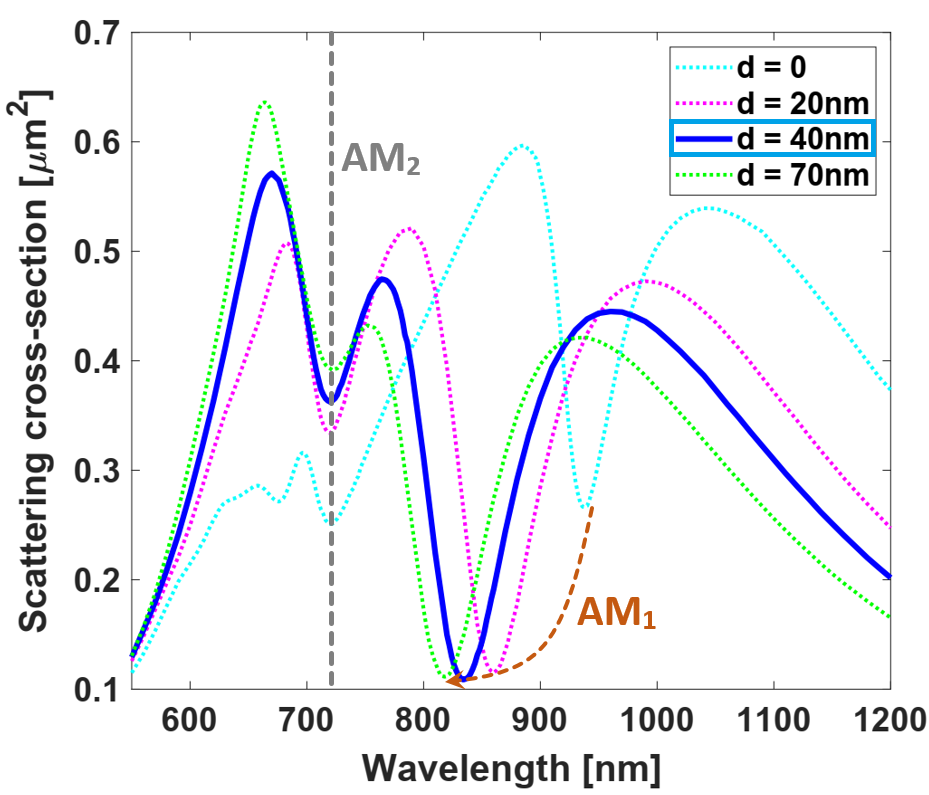


Fig. S2. Simulated total SCS of our proposed disk-based oligomer structure at normal incidence of plane wave versus wavelength for various deviation of corner particles denoted by$S=a+d$. Here, the size of the gaps is $g=8nm$ and the remaining structural and geometrical parameters are the same as those in Fig.2. Independent spectral tunability of the first-order anapole mode (AM_1_ mode) from the second one can be clearly observed.


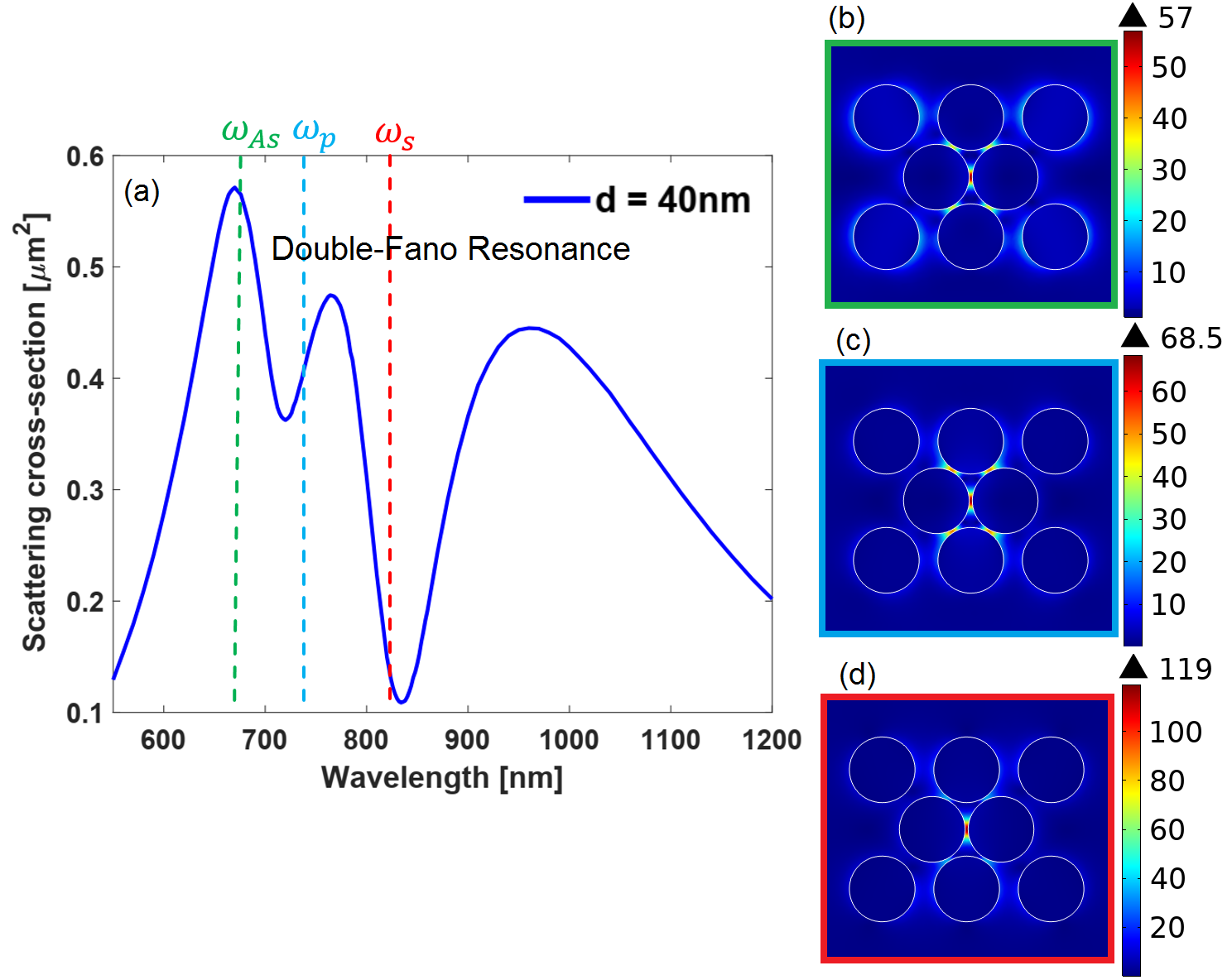


Fig. S3. (a) Simulated total SCS spectra of the disk-based oligomer $\left( R=84nm,h=20nm \right)$at normal incidence of plane wave for the gap distance of $g=8nm$ and with deviation value of$d=40nm$. Here the two Fano dips and a highly-scattering shoulder are just matched to the wavelengths of pumping (blue, 745 nm), Stokes (red, 820 nm) and anti-Stokes (blue, 682 nm) beams, respectively, providing a highly-efficient SECARS enhancement around 10^15^.

[1] Grahn, P., Shevchenko, A. & Kaivola, M. “Electromagnetic multipole theory for optical nanomaterials.” *New J. Phys.*14, 093033 (2012).

[2] Alaee, R., Rockstuhl, C. & Fernandez-Corbaton, I. “An electromagnetic multipole expansion beyond the long-wavelength approximation.” *Opt. Commun.* 407, 17–21 (2018).

[3] Garcia de Abajo, F. Javier. “Nonlocal effects in the plasmons of strongly interacting nanoparticles, dimers, and waveguides.” *J. Phys. Chem. C* 112.46 (2008): 17983-17987.
